# Philadelphia County Medical Society

**Published:** 1850-03

**Authors:** G. Emerson


### PHILADELPHIA COUNTY MEDICAL SOCIETY.

Dr. S. Jackson (late of Northumberland) in the Chair.

*Meeting for Discussion.*

(Reported by Mr. J. Aitken Meigs.)

*Upon the causes of the greater mortality of male children, and the influence operating to change the relative proportion of the sexes at birth.* By G. Emerson, M. D.

Up to the fifteenth year, there is an excess of 15 per cent. in the number of deaths of boys over that of girls. This excess in the male mortality is commonly ascribed to the greater exposure, and rougher sports and amusements of the boys; an erroneous idea, the fallacy of which is shown in the fact that the majority of the deaths of the males takes place in early infancy, when no such exposure and danger consequent to said rough sports can possibly exist. The deaths of boys, too, from climbing, swimming, &c., equal those of the girls from scalding, domestic accidents, &c.

The particular diseases which give rise to death in the two sexes, are very different in their nature and characteristics. Thus, males are attacked with violent inflammation of the brain, accompanied with serous effusions, convulsions, &c.; inflammations of the stomach, lungs and other important organs; while females suffer from hooping-cough, small-pox, measles, thrush, &c. In boys, the character of the disease is sthenic; in girls, asthenic. The diseases from which females suffer most are seated in the cutaneous and mucous tissues.

Of 100,000 deaths reported by the Registrar-general of England, 31,671 were under the fifth year; and of these, 15,006 were females, and 16,665 were males. Of the above, the number of deaths from inflammation of the brain were 2550 males, and 2081 females; of dropsy of the brain, 1481 males, 1151 females; small pox, 213 males, 240 females; hooping-cough, 1115 males, 1445 females; measles, 1048 males, 1028 females, etc.

From these and similar statistics, the inference follows that the disproportion in the deaths of the two sexes, during childhood, does not arise so much from exposure to external circumstances, as from differences in physical organization.

From the fact of boys succumbing so easily and so rapidly to diseases of a sthenic type, and females to those of an asthenic character, we deduce the practical hint of combatting most energetically the inflammatory symptoms, of the one, as soon as manifest, and preventing too great exhaustion of the system when symptoms of depression begin to appear in the female infants.

The Dr. then spoke of the effects of weather upon infant mortality, and more particularly of the limitation of the effects of hot weather, to the period of lactation. For interesting facts relative to this subject, he referred to statistics lately published by himself in the American Journal of the Medical Sciences.

During the first year of infant life, the season of the greatest mortality is the three hot summer months. The number 250 representing the mortality for May, we would have 836 as that for

July. After the second year the deaths are more equally distributed throughout the months; the number seeming even less in the hot than in the temperate and cold seasons. The heat, which at an earlier period was inimical, would now appear to be friendly to infantile life.

Dr. E. next referred to the influence of certain agencies which changed the ordinary proportions of the sexes. The general preponderance of males over females at birth, is about 7½ per cent. In 1833 the singular phenomenon of a reverse proportion was evident. There was not only a deficiency of male births, but moreover, in the months of April and May of that year, a decided female excess. Upon further investigations, this female excess was found to be the product of conceptions occurring in the months of August and September of 1832. This, as is well known, was the period of the first invasion of epidemic Cholera. Looking abroad for corroboration of this singular fact, it was found to hold good also, in the proportion of births occurring nine months after the epidemic had appeared at Paris. From this and other investigations, he arrived at the conclusion, that this change in the relative proportion of the two sexes at birth, was owing to the depressing influence of cholera. He has further observed that a tendency to the above result is always produced by the operation of any class of depressing agents, while circumstances that tend to high physical development increase materially the male excess.

In France and Prussia, where the mass of the people labor much harder than in our own country, and are poorly fed and clothed, the excess of female births is slightly under 6 per cent.; in England, 5 per cent.; in Philadelphia, 7.5 per cent.; and in our western country as high as 10 per cent.

Investigations into the comparative proportions of the sexes born in city and country populations, manifest the existence of a greater male excess in rural districts. This, from the foregoing observations, was to be expected, since in cities, intemperance, foul and vitiated atmosphere, unwholesome diet, and other depressing agencies, operate much more strongly than in the country. Hence, the Dr. observed, this proportion of the births of the two sexes, may be considered as a sort of natural thermometer of the physical comfort and advantages enjoyed by a community.

The institution of polygamy may have originated in a scanty supply of food occurring at some former period in the community where such institution exists, and evincing its depressing tendency by a predominance of the female over the male population. Once established it would foster itself.

The proportion of the two sexes being under such considerable control, it remains for the various legislative bodies throughout the civilized world to benefit and meliorate by their wise enactments the condition of the social cosmos.

Dr. J. Bell desired to suggest to Dr. E. an anatomical fact which he deemed would go far towards explaining the excess of male deaths. The male head, he stated, is larger at the period of birth than the female. and consequently much more liable to severe pressure during the active throes of labor; and as a consequence of such disastrous pressure of the cranial parietes upon the encephalic mass, death, or at least disease, insidiously, but surely terminating in death, must sooner or later supervene. In accordance with this anatomical observation, we find that the majority of male deaths occur during the early months of the first year of infantile life. Pregnant women, it is well known, die more frequently from giving birth to male infants, rather than female. The above facts Dr. B stated upon the authority of Dr. Simpson, by whom the observations in question—although not original with him—were brought before the profession. Dr. J. Clarke, of Dublin, had noticed the same fact, as had recently, Dr. Cheau, in an essay in the Annales d'Hygiène, et de Médicine Légale, Juillet, 1846. Tiedemann affirms, also, that the difference in the size of the male and female brain, at birth, is retained throughout life.

Dr. Darrach. The cerebral lobes of the male brain are larger than those of the female, while the cerebellum of the latter preponderates over that of the former. Hence the ovoidal form, which we so much admire, in the female head. During parturition, the male head, in consequence of its approximation to the spherical form, is subjected to an almost equal pressure upon either of its axes, while as respects the female ovoid, the difference between the pressure upon the conjugate and transverse diameter is considerable. This anatomical fact is sufficient to explain the greater mortality among the male infants after parturition. The seeming want of development of the cerebrum is amply compensated, in the female, by the proportionally larger size of the ganglionic system, and the more complicated nervous connections existing between the pelvic ganglia and viscera. The necessity and utility of this equilibrating preponderance in the sympathetic and pelvic ganglia, becomes obvious when we reflect upon the function which the female performs in the grand economy of life. Generation demands the continued exercise of her strongest sympathies, not only during the tedious, painful and laborious steps of the process, but long afterwards, when a living creature, weak and helpless, looks to her for sustenance, comfort and protection.

Dr. Emerson admitted that some weight might be due to the opinion, that serious injury was often sustained by the infant brain during parturition, but that this injury did not explain the cause of cerebral disease and mortality occurring at more advanced periods of infancy.

Dr. Coates desired to know why Philadelphia was pre-eminent, among all other cities in the Union, for the mortality that occurred in it from cholera infantum during the second year of infantile life.

Dr. Darrach. New York, Boston and Charleston enjoy sea air, while Philadelphia is enveloped almost uninterruptedly in an atmosphere maintained at a high temperature. Add to this the alimentary cause of irregular or improper diet, and we have efficient explanation of the origin of the cutaneo-abdominal symptoms, which are prominent in this disease. As confirmatory of this opinion, he mentioned the successful results which he had experienced in the treatment of the malady. Acting upon the suggestion of Dr. Chas. Caldwell, he was accustomed to send his little patients to Kaighn's Point, giving them, at the same time, calomel in minute doses. This place is selected as enjoying, from the peculiarity of its location, the benefits of a sea breeze above all others situated so high up in the course of the Delaware river. Old pilots and sailors, residents of this spot, bear testimony to the fact of the existence of a sea breeze, and speak of it in this respect as a sort of minor Cape May.

Dr. Emerson concurred with Dr. Darrach in thinking that the continued heat, such as we have in this city, uninterrupted by any or scarcely any refreshing coolness, was an efficient agent in producing the disease. But he would call to the minds of the gentlemen present the effects of such heat upon the inmates of our crowded courts and alleys especially, where the disease, in the majority of cases, originated, and was fostered by unwholesome food, a stifled and filthy atmosphere, &c. To the presence of refrigerating agencies in Boston, and some other ports upon the seaboard, he attributed their greater exemption from cholera infantum.

Dr. Parrish stated, that according to recent accounts, the mortality in St. Louis, from the disease in question, is greater even than in Philadelphia. In reference to the malady as existing in Philadelphia, the Dr. desired to state, that in addition to the causes already advanced by the gentlemen present, the mode of building in courts and alleys, and immediately opposite blind walls, was such as would tend to keep up the disease continually. Such buildings, moreover, were constantly being erected, even in the recent portions of the town. As far as he was aware, this cause did not exist so extensively in New York, or any other of the places mentioned during the evening's discussion.

Again, the custom of causing the child to sleep between the parents, is of itself sufficient, during the hot nights of our summer, to bring on an attack of the disease. In relation to Dr. Darrach's hint at the practical treatment of the disease, he declared the practice to be an old one, and that Dr. S. P. Griffiths was in the habit of lancing the gums merely, and sending the little sufferers into the country. Dr. P. affirmed himself to be a living example of the propriety of such practice. When young he was attacked with the disease, and being brought to the verge of the grave, his father resolved as a dernier resource to try the effects of a change of locality. Placed upon a steamboat and borne rapidly away over the bosom of the Delaware; but a few hours sufficed for the fresher atmosphere he then inspired, to invigorate his whole system, and breathe anew into him, as it were, "the breath of life." From that moment he rapidly recovered.

Dr. Darrach, regarded solar influence as the efficient cause of the complaint in our city, unabated as it is by the sea breeze which daily blows through the crooked lanes and alleys of New York, robbing them greatly of their heat.

Dr. Jackson inquired of Dr. D. if narrow streets and alleys were so much warmer than broader thoroughfares; if they were not in reality cooler. Nero, it should be recollected, in rebuilding the city of Rome, after its conflagration, having widened the streets, and enlarged the public places of meeting, was inveighed against by the citizens as having let in the sun-light to scorch them, so much warmer had the city become.

Dr. Darrach remarked in reply, that while Paris, with its narrow streets and lofty houses,—the latter shielding the former from the sun,—was comparatively cool, Washington with its broad streets was proverbially uncomfortable, in consequence of the heat reflected from the houses.

Dr. H. S. Patterson, from Dispensary and other experience, had been induced to believe, that the disease in question was not so common among the colored as among the white population of our city ; and that among those Irish emigrants who were exposed to the same exciting causes, the mortality from the disease was greater than among any other class of the people. Improper diet, placing the children early at the table, the hard labor continually undergone by the mother, and the unwholesome food upon which she habitually fed, he considered as the predisposing causes exceedingly active in the production of the complaint among the negroes and the destitute whites. Whether, upon the one hand, the facility, with which the negro repels the malady, is purely the effect of a constitutional peculiarity, transmitted to him by his African ancestors ; and whether the sudden and continued vicissitudes of our climate, so unlike the comparatively equable seasons of Ireland, constitute the efficient cause of the greater mortality among the Irish, are questions of deep interest as regards the etiology of the disease. Upon this point, and also whether cholera infantum was on the increase in the neighboring country towns, he desired information of the gentlemen present, inasmuch as it was a matter of practical utility to determine the propriety of sending patients to the surrounding country for relief.

Dr. J. Bell, in confirmation of the influence of high temperature in producing cholera infantum, adverted to the great mortality in London, the year before last, from this disease, owing to the unusually intense heat of the summer. He regarded fresh air, and pure Schuylkill water, as having done more for the prevention of the disease, in this city, than any other two causes. In reference to the neglect of suitable hygienic measures elsewhere, he stated that in fifty towns in England in which the disease was rife, six only were fairly, not fully, supplied with water, and had a proper amount of sewerage. He also animadverted at some length upon the imperfect ventilation which existed in the houses of the rich, owing to the erroneous plans of building of the present time.

Dr. Darrach desired to make an observation, having some bearing upon Dr. Patterson's remarks. According to statistics which he had kept while physician in the Eastern Penitentiary, it was found that during the winter season, the negro prisoners, principally, were affected, and with chronic pleurisy; while during the summer, the white prisoners, with diarrhœa.

Dr. Jackson remarked that negroes generally were more commonly affected with disease of a bilious remittent type, to which cholera infantum was supposed to be closely allied.

